# Anti-IL5 therapy for asthma and beyond

**DOI:** 10.1186/1939-4551-7-32

**Published:** 2014-12-04

**Authors:** Manali Mukherjee, Roma Sehmi, Parameswaran Nair

**Affiliations:** St Joseph’s Healthcare & Department of Medicine, Firestone Institute for Respiratory Health, McMaster University, 50 Charlton Avenue East, Hamilton, Ontario L8N 4A6 Canada

**Keywords:** Eosinophil, IL-5, Eosinophilic asthma, Hypereosinophilic syndrome (HES), Churg-strauss syndrome, Chronic bronchitis, Eosinophilic granulamatosis and polyangitis (EGPA), Chronic obstructive pulmonary disorder (COPD), Mepolizumab, Reslizumab, Benralizumab

## Abstract

Airway inflammation is considered to be the primary component contributing to the heterogeneity and severity of airway disorders. Therapeutic efficacies of diverse novel biologics targeting the inflammatory pathways are under investigation. One such target is IL-5, a type-1 cytokine that is central to the initiation and sustenance of eosinophilic airway inflammation. Over the past decade, anti-IL5 molecules have been documented to have mixed therapeutic benefits in asthmatics. *Post hoc* analyses of the trials reiterate the importance of identifying the IL-5-responsive patient endotypes. In fact, the currently available anti-IL5 treatments are being considered beyond asthma management; especially in clinical complications with an underlying eosinophilic pathobiology such as hypereosinophilic syndrome (HES) and eosinophilic granulomatosis and polyangitis (EGPA). In addition, closer analyses of the available data indicate alternative mechanisms of tissue eosinophilia that remain uncurbed with the current dosage and delivery platform of the anti-IL5 molecules.

## Introduction

The past ten years have witnessed the development and evaluation of a number of biologics that target the Th2 cytokines involved in asthma pathophysiology, particularly those that are associated with eosinophils in the airway. Eosinophils play a key role in the pathobiology of several airway disorders presenting with chronic inflammatory pathology such as asthma [1, 2], chronic obstructive pulmonary disorder (COPD) [3], eosinophilic granulamatosis and polyangitis (EGPA) [4], and hypereosinophilic syndrome (HES) [5]. Targeting Interleukin-5 (IL-5) in asthma, the central protagonist in eosinophilia (discussed in details later), was a logical derivative post promising results in animal models [6, 7] and initial screenings in patients [8, 9]. Deliberation arises from the mixed response of anti-IL-5 trials conducted in different asthmatic populations that document a healthy reduction in circulating eosinophils, but without much significant improvement in other clinical indices of disease severity (extensively reviewed in [10–12]). Again in a recent review, anti-IL-5 therapy has been conjectured to be effective in long-term management of HES patients [5]. The current review will critically evaluate the documented outcomes of the conducted clinical trials to date and subsequently assess the therapeutic implications of anti-IL-5 therapy in treating airway disorders with an aberrant eosinophilic pathobiology.

### Eosinophil biology and the role of IL-5

**Figure 1:**
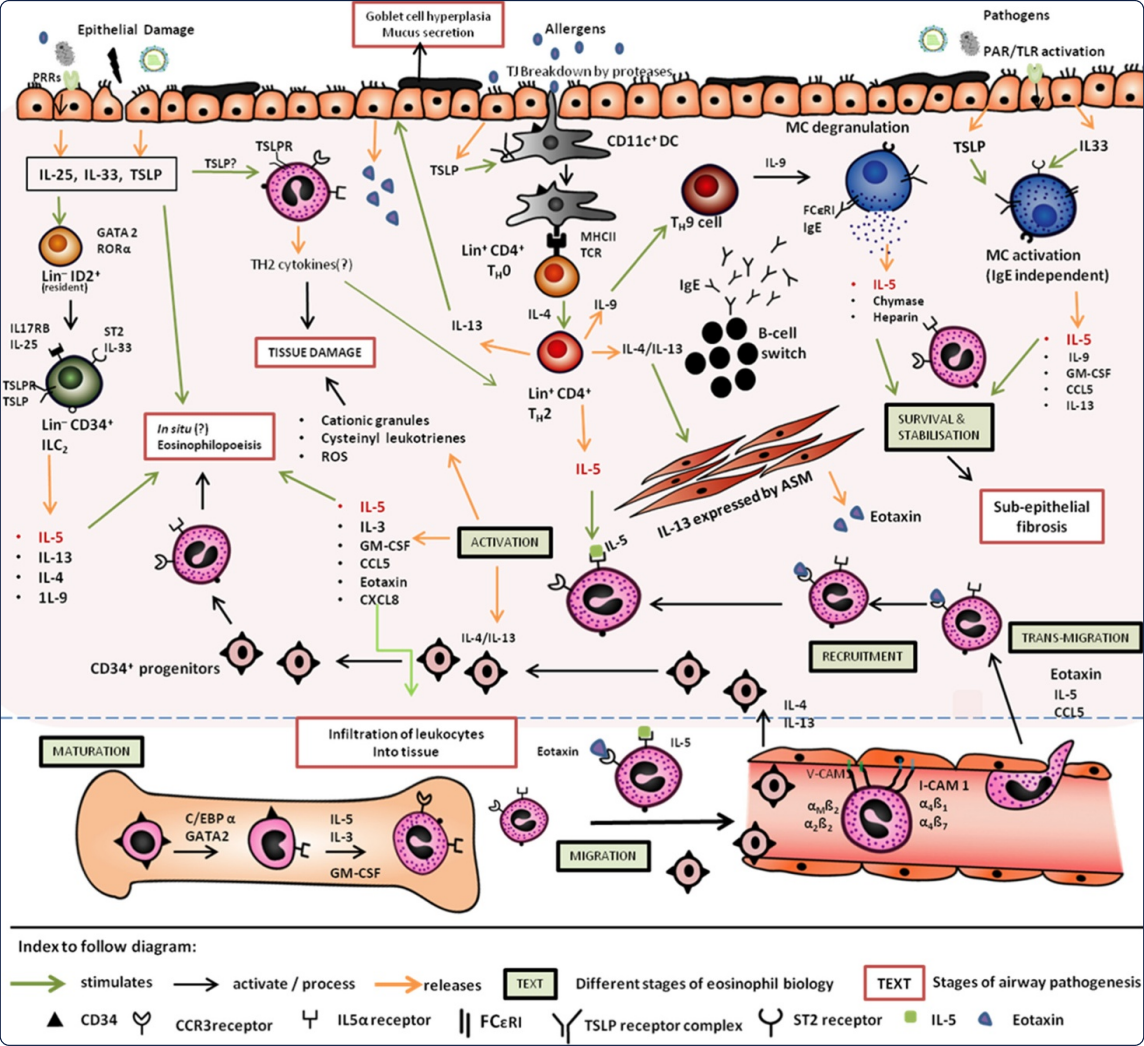
**A schematic representation of eosinophilia in the airways.** The figure portrays **(A)** the complex eosinophil biology: *Maturation*: CD34^+^ myeloid progenitor cells (bone-marrow) differentiate into the IL5α^+^ CCR3^+^ eosinophil-committed progenitor cells under the influence of the different transcription factors like GATA2 and C/EBPα. IL-5, IL-3 and GM-CSF stimulate their further maturation into eosinophils. *Migration*: release into the circulation is coordinated synergistically by IL-5 and eotaxin. *Transmigration*: under the influence of IL-5 and eotaxin, the eosinophils ‘seep’ out through the endothelium. *Recruitment:* Eosinophil trafficking into the site of inflammation is selectively regulated by IL-5, eotaxin and CCL5, in addition to a multitude of cytokines. *Activation*: IL-5 binds to IL-5Rα and activates eosinophils to release a multitude of cytokines, eosinophilic granular proteins, cysteinyl leukotrienes, that lead to tissue damage and further aggravates the inflammatory process. *Survival* and stabilisation: IL-5 released from different sources and products from mast cell (MC) degranulation suppresses apoptosis and allows survival of eosinophils in the submucosa. **(B)** Different sources of IL-5 (in red) and sustenance of eosinophilia: (i) the canonical T_H_2 pathway initiated by dendritic cell (DC) activation releases IL-5. (ii) MC activation is another source of IL-5 that can be triggered by IgE binding to the FCϵRI receptor or by epithelial-derived Type 2 alarmins like TSLP and IL33; or via T_H_9 pathway (iii) Type-2 alarmins (IL-33, IL-25, TSLP) can activate the lineage negative ID2^+^ lymphoid cells resident in the tissue to differentiate into lineage negative ILC_2_s that can release IL-5 and IL-13, and drive eosinophilic inflammation (iv) IL-13 and IL-4 can recruit CD34^+^ progenitors cells from bone marrow into the lung tissue where it can differentiate into eosinophils in presence of IL-5. *N.B. Diagram is not up to scale. Mechanisms relevant to only eosinophilic inflammation has been included.*

A robust literature now support eosinophils to be pleiotropic, multifunctional leukocytes that facilitate the ‘innate’ response against extraneous agents in the airway, modulate the downstream ‘adaptive’ immunity cascade, maintain local immunity/inflammation and as the end-stage effector cells that can cause tissue damage *via* release of granule proteins, reactive oxygen species and cysteinyl leukotrienes (reviewed in [2, 13]). In eosinophil biology (refer to Figure 1), IL-5 plays a central role in the production, mobilization, activation, recruitment, proliferation, survival and suppression of apoptosis in eosinophils at the site of inflammation (reviewed in, [2, 6, 12, 13]), illustrated in a schematic diagram (Figure 1).

In 1996, a study reported that ‘IL-5 deficient’ mice failed to develop the characteristic eosinophilia and airway hyper-reactivity after ovalbumin-sensitization [7]. Soon after, 8 asthmatic patients demonstrated increase in airway eosinophil counts and methcholine PC_20_ (a provocative concentration of methcholine required to induce 20% reduction in the forced expiratory volume in 1 second, FEV_1_) when subjected to inhaled recombinant IL-5 [9]. Moreover, restraining sources of eosinophil recruitment and/or eosinophil-deficient animal models were observed to be healthy without any characteristic abnormalities [14]. As a logical derivative from the existing experimental and clinical evidences, several monoclonal antibodies (mAbs) were engineered to neutralize free circulating IL-5 and/or target IL-5 receptor alpha (IL5Rα) and are now in different phases of development [10, 12, 15].

### A comparative analysis of the Anti-IL-5 trials: asthma

As early as 1990, Bousquet et al., [8], correlated eosinophilia with asthma severity and demonstrated eosinophilic cation proteins (ECP) were associated with epithelial damage in 44 patients with asthma. A direct but modest correlation has been established between asthma severity, frequent exacerbations and the intensity of eosinophilia. Thereby, a sub-set of patients are being identified who suffer from ‘severe refractory asthma’, consequently accounting for a high socio-economic burden and are considered to most benefit from an eosinophil-targeted therapy [1, 10].

The last 15 years have documented several clinical trials that evaluate the therapeutic relevance of anti-IL-5 biologics in asthma treatment and symptom management (refer to Table 1). As evident from the outcome summary tabulated in Table 1, Mepolizumab, a humanized mAb (IgG1) with a high affinity for binding free IL-5 (which further prevents its binding to the receptor, IL5Rα), is found to be effective in depleting eosinophil numbers in blood and the airways. On the contrary, studies in mild-moderate asthmatics documented Mepolizumab to be ineffective in improving end-point clinical symptoms (refer to Table 1, [16, 19, 20]), therefore raising concern over the efficacy of IL-5 as a therapeutic intervention in asthma. However, by selecting patients with persistent blood ( >0.3 × 10^9^/L) and sputum eosinophils (≥3%) coupled with frequent history of exacerbations, two independent relatively small studies in 2009 documented a significant decrease in the exacerbation frequencies (P ≤ 0.02 *vs.* placebo, both studies) and asthma control questionnaire (ACQ) scores (P ≤ 0.02, *vs.* placebo, both studies), with 750 mg infusions of Mepolizumab [21, 22]; in addition to a prednisone-sparing effect [21]. Similar reduction in exacerbation frequencies with corresponding decrease in peripheral blood eosinophils was reflected in a large, double-blinded, placebo-controlled, multi-centered study conducted in 2011 [23]. Based on the dose–response observations from the DREAM study [23], 75 mg intravenous and 100 mg subcutaneous doses were investigated in a recent Phase III trial, where significant reductions in exacerbation rates by 47% and 53% respectively (P< 0.001, *vs.* placebo) along with depletion of blood eosinophils were recorded [28]. The 100 mg subcutaneous dose was reported in a parallel Phase III study to have a corticosteroid-sparing effect in a similar target population, with median percentage reduction of 50% in treatment group, along with 32% relative reduction in annual exacerbation rate (p= 0.04 *vs* placebo) [29]; a reduction though statistically significant, is less pronounced than that observed in the earlier study with higher dose and intravenous route of drug delivery [21, 32]. The optimum dose, route and duration of therapy and persistence of beneficial effects for prednisone-dependent patients remain to be established.

**Table 1:** **A comparative study of Anti-IL5 trials in Asthma**

First author [ref] year/ Drug	Disease (severity)	Study design	Dosage/ delivery	Inclusion criteria: Baseline eosinophil count	Comments on eosinophilia	Outcome summary
Leckie [16] 2000	Mild atopic asthmatic	n= 24 mc, db, pc	Single dose i.v., 2.5, 10 mg/kg	• Not an inclusion criteria	• Day 29, post-allergen 10 mg/kg dosage, blood eos 0.04 × 10^9^/L compared to 0.25 × 10^9^/L placebo (P< 0.0001)	• No significant effect on AHR
**Mepolizumab**	FEV_1_ ≥ 70%, predicted			Baseline values:		• No significant effect on late asthmatic response to allergen challenge
				•Sputum eos (% mean) > 11% in all groups		
					• Day 29, post- allergen,10 mg/kg dosage: 0.7% sputum eos compared to 12.8% placebo ( p= 0.005)	
				• Blood eos (counts × 10^9^/L) > 0.2 in all groups		
Bȕttner [17] 2003	Mild to moderate asthmatics	n= 19 mc, db, pc	Three monthly doses, i.v.	No Baseline count/ median n/a	• Decrease in blood eos (median values from 300 to 45 per mL, P< 0.05 *vs.* placebo)	• No asthma end-points were assessed
**Mepolizumab**	FEV_1_ > 50-80%, predicted		250/750 mg			• T-cell sub-sets and T-cell cytokine levels not altered
					• Decreased levels of serum ECP (median values from 15 to 5 mg.L^−1^, P< 0.05 *vs.* placebo)	
						• No sputum data
Kips [18] 2003	Moderate-severe asthma, FEV_1_ > 40-80%, predicted	n= 32 db, pc, mc	Rising single dose (0.03, 0.1 , 0.3, 1 mg/kg) i.v	• Not included in the inclusion criteria	• Dose dependently reduced circulating eos	• Significant increase in FEV_1_ post 24 hours from dose range ≥ 0.3 mg/kg (p= 0.019)
**Reslizumab**				Baseline value:	• Significant dose reduction with 1 mg/kg for 30 days post dosing ( p=0.05)	
				• blood eos (counts × 10^9^/L):		• No significant changes in other clinical indices
				Placebo:0.45 ± 0.16	• No significant trend in changes of sputum eos were observed between groups due to the wide variability in baseline counts between the groups	
				0.3 mg/kg : 0.28 ± 0.04		
				1.0 mg/kg : 0.25 ± 0.04		
				• Sputum eos (% mean)		
				Placebo:22.9 ± 12.5		
				0.3 mg/kg : 2.6 ± 0.44		
				1.0 mg/kg : 5.5 ± 3.92		
Flood-page [19] 2003	Mild atopic asthma	n= 24 db, pc, parallel-group,	3 i.v. doses of 750 mg	• Not included in the inclusion criteria	• Blood eos: significant reduction in wk 4 and 10 (P<0.02, *vs.* placebo)	• Sputum eos not checked
**Mepolizumab**	FEV_1_ ≥ 70%, predicted		Mepolizumab/ per month 12- wk follow up	Baseline value:		• No change in clinical parameters, FEV1, AHR
				• Blood eos (mean × 10^9^/L):	• Bone marrow: 70% reduction in mature eos (P= 0.017)	
				Group: 0.27		
				Placebo: 0.4	• BAL fluid eos: median reduction of 79% from baseline (P= 0.4 *vs.* placebo) ns	
Flood-page [20] 2007	Moderate persistent asthmatics	n= 362 mc, db, pc	3 i.v. doses of	• Not included in the inclusion criteria	• Blood eos: Sustained significant 80% reduction for both doses ( p< 0.001 *vs.* placebo)	• No significant change in clinical end-points
**Mepolizumab**	FEV_1_ ≥ 50-80%, predicted		750/250 mg Mepolizumab per month	• Baseline blood eos for all group showed median values ≥ 0.3 × 10^9^/L	• Sputum eos significant reduction from baseline (P=0.006, 250 mg, P= 0.004, 750 mg)	• Trend for a reduction in exacerbation rate, ns
						• Decrease in summary symptom score *vs.* placebo for 750 mg at wk 12 (P= 0.032)
			8- wk follow up			
Nair [21] 2009	Severe persistent asthma with Eosinophilia	n= 20	5 i.v. doses of 750 mg per month.	• Yes. Inclusion criteria - Sputum eos > 3%	• Significant reduction in blood eos after 1st dose (49.5/μl), last dose (64.5/μl) and follow up (76.3/μ) (P< 0.05) vs. placebo, no significant reduction from baseline	• Significant reduction in asthma exacerbations with drug (1) compared to placebo (12 in 10 patients), P< 0.01
**Mepolizumab**		db, pc, pilot study				
	FEV_1_%,predicted value (median ± SD): 48 ± 17 (drug) 52 ± 13 (placebo)		Prednisone dosage tapered after 2^nd^ infusion	Baseline:		
				• Blood eos;		• 83.8% reduction in prednisone dose *vs.* placebo (P< 0.04)
				Drug: 664 ± 492.5/μl; placebo:352 ± 253.7/μl	• Significant reduction in sputum eos after 1^st^ dose (0%), last dose (1.3%) and follow-up (0.3%) (P< 0.05) *vs.* placebo, no significant reduction from baseline	
						• FEV_1 -_ significant improvement *vs*. placebo, P< 0.05
				• Sputum eos:		
				Drug group: 16.6%		• ACQ: significant improvement from baseline P= 0.01, *vs.* placebo
				Placebo: 4%		
Haldar [22] 2009	Refractory eosinophilic severe asthma	n= 61 db, pc, parallel study	12 doses of 750 mg i.v. per month	• Inclusion criteria - Sputum eos > 3%	• Blood eos: reduced by a factor of 6.6 from baseline in drug group, compared to 1.1 in placebo (P<0.001)	• Reduction in number of exacerbation over the course of 50 wks (P= 0.02)
**Mepolizumab**				Baseline:		
				• Blood eos (x 10^9^/L);		• AQLQ score increase with drug (P= 0.02, *vs.* placebo)
				Drug:0.32 ± 0.38 placebo: 0.35 ± 0.30	• Sputum eos: reduced by a factor of 7.1 from baseline in drug group, compared to 1.9 in placebo (P=0.002)	
				• Sputum eos:		• No significant difference in group in AHR, FEV_1_, ACQ
				Drug: 6.84 ± 0.64%		
				Placebo:5.46 ± 0.75%		
Pavord [23] 2012	Severe refractory asthma with ≥ 2 exacerbations in past year	n= 621	3 doses s.c., at 4 wks	• Yes. Inclusion criteria - Sputum eos > 3%	• Blood eos (x10^9^/L): at 52 wk, *vs.* placebo	• Exacerbation rates at all doses were 39-52% less than those in the placebo group (P< 0.05 *vs.* placebo)
**Mepolizumab**		db, pc, parallel study, mc	75/250/750 mg		75 mg: 0.22< 0.0001, 250 mg: 0.14 p< 0.0001	
		(DREAM)	52 wk	Blood eos ≥ 0.3 x10^9^/L	750 mg:0.12 p< 0.0001	
				Baseline:	• Sputum eos (ratio): at 52 wk	• No changes in FEV_1_, ACQ, AQLQ
				• Blood eos (x 10^9^/L);	75 mg : 0.68, ns	• Lowest dose of 75 mg was near to the top of the dose response curve w.r.t reduction of blood eosinophils
				>0.2 , for all groups	250 mg: 0.35, ns	
				• Sputum eos:	750 mg :0.12, p= 0.0082	
				>6% for all groups		
Castro [24] 2011	Poorly controlled asthma, on high dose ICS	n= 106	3.0 mg/kg sc, at baselineand at Weeks 4, 8, and 12	• Yes. Inclusion criteria - Sputum eos > 3%	• Significant reduction in blood eosinophils (P< 0.0001, *vs.* placebo)	• Trend in reduction of asthma exacerbations in drug group (p= 0.083, ns)
**Reslizumab**		db, pc, parallel study,				
				Baseline:	• 95.4% reduction in sputum eos compared to placebo, 38.7% (p= 0.0068)	• ACQ trend in favour of drug group (p=0.054)
				• Blood eos , median (x 10^3^/μL);		• Significant improvement in ACQ score in patients with nasal polyps (p= 0.012)
				Drug: 0.5		
				Placebo: 0.5		• Significant reduction in FEV_1_ in drug group (p=0.002, *vs.* placebo)
				• Sputum eos (%):		
				Drug: 10.7		
				Placebo: 8.5		
Busse [25] 2010	Mild atopic asthma	n= 44 mc, safety in open-label study	Single escalating doses (0.0003-3 mg/kg, over 3 – 30 minutes)	No. this was a safety study.	• Significant decrease in eos in dose-dependent fashion from baseline to 0.01 ± 0.0 × 10^3^/μL, 24 hours post-dose	• Acceptable safety profile
**Benralizumab (MEDI-563)**	FEV_1_ ≥ 80% of predicted			Baseline:		• No adverse reactions were noted.
				• Blood eos:		
				Mean ± SD, 0.27 ± 0.2 × 10^3^/μL	• 94% patients on doses ≥03.mg/ml showed 0–0.1 × 10^3^/μL blood eos.	
				• ECP levels (mean)		
				21.4 ± 17.2 μg/L	• ECP levels were reduced from baseline to 10.3 ± 7.0 μg/L, 24 hrs post-dosing	
Laviolette [26] 2013	Eosinophilic asthma	n= 27 mc, db, pc	• Cohort 1 – (i.v) 1 mg/kg single dose	• Sputum eosinophil counts of ≥2.5%	• Significant reduction in sputum eosinophils, airway eosinophil counts and 100% reduction in bone marrow and peripheral blood	• Additional clinical factors were not measured
**Benralizumab**	FEV_1_ ≥ 65%, predicted			Baseline:		
			• Cohort II-100 mg, 200 mg, combined 3 monthly (s.c).			
				• Sputum eos (mean%)	• Airway mucosal/submucosal eos: mean reduction *vs*. placebo:	
				• Cohort 1:		
				Placebo : 13.9	Cohort I : (i.v.) 61.9% (ns)	
				Drug: 6.6	Cohort II, combined (sc): 83.1% (p= 0.0023)	
				• Cohort II:	• Induced sputum eos (mean% )	
				Placebo: 34.1	Cohort I: 4.5%, day 21 compared to 20.8% placebo	
				100 mg: 10.5		
				200 mg: 4.9	Cohort II: (combined) 0.6% at day 28, compared to 6.4% placebo	
				Combined: 7.4		
Castro [27] 2014	Uncontrolled asthma ACQ-6 score ≥ 1.5	n= 609	• 2 mg, 20 mg, 100 mg sc for eosinophilic patients (n= 324)	• Subjects were stratified based on blood eos, Sputum eos ≥2%, FeNO > 50 ppb	• All doses reduced blood eos<50 cells/μl after the first dosage	• Significant improvement in FEV_1_ and ACQ-6 in eos subtype with all doses
**Benralizumab**		(324 – eosinophilic, 282)				
	Exacerbation ≥ 2/last year				• In eosinophilic group, 100 mg sc improved annual exacerbation rate by 41% (p= 0.096) vs. placebo, deemed significant; ns in non-eosinophilic group,	• High incidence of adverse reactions in treatment arm
		Phase IIb	• 100 mg sc for non-eosinophilic (n= 282)			
		Db, pc, dose-ranging study				
			• 7 doses every 4 weeks			
					• Subgroup analysis showed greater improvement with increased baseline blood eos (100 mg sc reduced exacerbations by 70% in patients ≥ 400 cells/μl, p= 0.002)	
Ortega [28] 2014	Severe asthma	n= 576	• Cohort 1 – 75 mg i.v. (n= 191)	• Blood eos 150/μl at screening or 300/μl in previous year	• Reduction in eos by week 4 mainted through the entire study	• Rate of exacerbations reduced by 47% and 53% in s.c and i.v. groups respectively (p< 0.001, *vs.* placebo)
**Mepolizumab**	Recurrent exacerbations, with ≥2 in previous year	mc, db, pc				
		Phase III	• Cohort 2 – 100 mg s.c. (n= 194)	• No sputum eos were accounted	• 83% reduction in i.v. group	
	ICS dose ≥880 μg fluticasone propionate				• 86% recution in s.c. group (p< 0.001, *vs.* placebo)	• Improvement in FEV_1_ for both groups (p< 0.05) and asthma scores (p< 0.001)
			Every 4 weeks for 32 weeks			
Bel [29] 2014	Severe eosinophilic asthma	n= 135 mc, db, pc	• 100 mg s.c. every 4 weeks for 20 weeks	• Inclusion criteria did not account sputum eos	• Drug significantly reduced blood eos by week 4 and was maintained throughout study (p< 0.001)	• Median percentage decrease in OCS from baseline - 50% in drug arm to no reduction in placebo (p= 0.007)
**Mepolizumab**	On 5–35 mg of daily OCS, and severe exacerbations					
		Phase III		• Blood eos 150/μl at screening or 300/μl in previous year		
						• Relative reduction of 32% in annual exacerbation rate despite lowering of OCS in drug arm (p= 0.04, vs. placebo)
						• Improvement in ACQ-5 score (p= 0.004)
Corren [30] 2014	Moderate-severe asthma	n= 395 (drug)	• 3.0 mg/kg, i.v., monthly (for 16 weeks)	• Inclusion criteria doesnot include sputum eos	• Abstract does not document any reduction in blood eos	• Significant reduction in ACQ score in drug arm (p= 0.04)
**Reslizumab**	ACQ ≥ 1.5	n= 97 (placebo)				
	On medium dose ICS (~440 μg fluticasone)	db, pc, mc, Phase III		• Study population stratified by baseline blood eos ≥ or ≤ 400 cells /μl	• Only 20% of study population was eosinophilic (or ≤ 400 cells /μl)	• FEV_1_ improvement for overall population by 68 ml, 270 ml for eosinophilic patients (p= 0.04 *vs.* placebo), ns increase of 33 ml in non-eosinophilic patients
Bjermer [31] 2014	Eosinophilic asthma	n= 311 db, pc, parallel	• 0.3 – 3.0 mg/kg, i.v., monthly (for 16 weeks)	• blood eos ≥ 400 cells /μl	• eosinophil measurement was not documented in the abstract	• overall improvement in FEV1 p ≤0.024, ACQ score (p ≤ 0.03)
**Reslizumab**	ACQ ≥ 1.5			• sputum eos not accounted		
		Phase III				• Higher dose - significant FEV_1_ increase as early as 4 weeks

Index: eos= eosinophils; db= double-blind; pc= placebo-controlled; mc= multi-center; sc= single-centre; FEV_1_= peak expiratory flow i.v= intravenous; s.c.= sub-cutaneous; wk= week; ns= non-significant; ACQ= Asthma Control Questionnaire, ICS= inhaled corticosteroid, OCS= oral corticosteroid.

Another anti-IL5 mAb (IgG_4/k_) Reslizumab, showed similar reduction in sputum eosinophils, significant improvement in lung function (P= 0.002, *vs.* placebo) and a trend towards improved asthma scores (P= 0.054, *vs.* placebo) in patients diagnosed with severe refractory eosinophilic asthma (see Table 1). Additionally, the authors observed the improvement in ACQ scores were most pronounced in patients with nasal polyps (P= 0.012, *vs.* placebo), [24] which reflected the observations of Gevaert et al., in 2003 [33]. There are recent reports of Phase III trials that demonstrate significant improvement in ACQ scores and FEV_1_ (p<0.05, *vs.* placebo) in moderate to severe asthmatics treated with 3.0 mg/kg of intravenous Reslizumab; charting a larger improvement in asthma control for subjects with baseline eosinophils ≥400 cells/μl [30, 31].

IL5Rα expressed by both mature eosinophils and eosinophil-lineage progenitor cells [2], is targeted by Benralizumab (MEDI-563), a humanized, afucosylated mAb. Being afucosylated, this drug induces apoptosis in its target cells *via* enhanced antibody-mediated cellular toxicity (ADCC), and is considered to have an increased efficiency of eosinophil depletion comparative to the other anti-IL5 biologics [34]. The initial safety trial conducted by Busse *et al.,* in 2010, documented no adverse events [25] and a further study by the same group showed 100% reduction of peripheral circulating eosinophils (Table 1) [26]. More recently, 100 mg subcutaneous Benralizumab exhibited significant improvement in annual exacerbation rates, lung function and asthma score, with greater benefits seen in patients with blood eosinophil levels ≥ 400 cells/μl [27]. Currently, there are three clinical trials registered on http://clinicaltrials.gov (NCT01914757, NCT02075255, NCT01928771, last accessed 17/09/2014), where the drug is being assessed as an adjunct therapy for ‘uncontrolled’ asthma.

### Further assessment of anti-IL5 Trials: clinical insights

The mixed outcomes from anti-IL-5 clinical trials highlight the need for careful endotyping of patients, since the therapy is deemed effective on those patients whose asthma is dependent on the eosinophilic inflammatory pathway [1]. The potential ‘responders’ to IL-5 therapy are patients who present with eosinophilia (blood >0.3 × 10^9^/L, >3% sputum), are generally steroid-responsive, and suffer from frequent exacerbations. As evident from the DREAM study [23], the atopic status is inadequate for segregating ‘responders’ from the ‘non-responders’, since approximately 50% of the patients who responded to Mepolizumab had negative radioallergosorbent test to the four most common allergens. In addition, sub-sets of patients that are aspirin-sensitive/induced asthma or present with sinusitis might also benefit from anti-IL5 therapy [35].

Using ‘sputum eosinophils’ as a biomarker to identify IL5-treatment responsive patient-groups as well as a marker for its therapeutic outcome is a topic under debate [1]. Studies that considered patients with ≥ 2.5 - 3% sputum eosinophilia in their inclusion criteria, independently recorded significant improvements with asthma scores and lung function [21, 22] compared to others (see Table 1). Similarly *post hoc* analysis showed that patients treated with 1.0 mg/kg Reslizumab, with baseline sputum levels< 3% did not show improvement in the FEV_1_, (even with depleted peripheral eosinophil levels) [18]. In fact, sputum eosinophils do not correlate with a change in circulating eosinophil numbers in the severe prednisone-dependent asthmatic patients and the former is markedly reduced before an event of exacerbations [1].

Subcutaneous doses of Mepolizumab showed neither significant reduction in sputum eosinophils (for 75 and 250 mg dosage groups) nor any relevant improvements in the symptom scores or lung function (see Table 1, [23]). The recent phase III trials documented depletion of blood eosinophils, significant improvement in asthma symptom scores and moderate reduction in exacerbation rates with lower (100 mg) subcutaneous doses (refer to Table 1), without any indication of whether the luminal eosinophilia generally exhibited in the specific patient group was resolved or not [28, 29]. In contrast, 750 mg intravenous infusions in the previous two studies of similar disease profiles [21, 22], were able to reduce both circulating and sputum eosinophils, allow significant improvement in ACQ, FEV_1_ and quality of life score along with pronounced reduction in exacerbations. This discrepancy may reflect the therapeutic significance of the drug delivery platform and dose used, a concern addressed in a recent editorial [32].

### Further assessment of Anti-IL5 trials: molecular insights

The eosinophil biology is complex and outcomes from the anti-IL-5 clinical trials reiterate this. Many of the anti-IL-5 clinical trials (Table 1) document the presence of tissue eosinophilia in spite of nil/low circulating levels, post-treatment. Especially, both studies with Benralizumab showed 100% reduction of eosinophils in bone-marrow and peripheral blood, but presence of airway mucosal/sub-mucosal eosinophils [26] and detectable levels of ECP in the sputum [25], indicating an alternative mechanism to IL-5 for eosinophil initiation, recruitment, activation and survival in the tissues. Delving further, Haldar et al., [22] showed significant decrease (P< 0.002) for both circulating blood and sputum eosinophils (see Table 1) in the Mepolizumab study group, which was not reflected in the paired bronchial-biopsy specimens (obtained before and after the study). In context, antisense oligonucleotide therapy (TPI ASM8), developed to suppress the expression of surface receptors CCR3 (C-C chemokine receptor type 3, binds eotaxin) and β chain (shared receptor for IL-5, IL-3 and granulocyte macrophage-colony stimulating factor, GM-CSF) [36], reduced sputum eosinophil counts by 46%; while a CCR3 antagonist was recently documented to show no effect of blood or sputum eosinophilia or to have any clinically improvement in moderate to severe asthmatics [37]. Scattered evidences [2, 11, 38] instrument the presence of alternative pathways *in situ,* that can trigger, activate and maintain eosinophils in the sub-mucosal and mucosal surfaces, independent of the classical T_H_2 pathway activation triggers (refer to Figure 1).

Extraneous environment-derived factors including non-allergic sources like pathogens and epithelial damage can trigger the release of epithelium-derived ‘Type-2 alarmins’ – IL-25, IL-33, and thymic stromal lymphopoeitin (TSLP, see Figure 1) [15, 38]. IL-25 and IL-33 can initiate mast cell (MC) response (mostly sub-mucosal localization) that leads to the release of IL-5 and CCL5. In addition, the type-2 alarmins activate the resident lineage negative, type 2, innate lymphoid cells (ILC_2_s) to release the classical T_H_2 cytokines IL-5, IL-13, IL-9, that directly or indirectly support eosinophil recruitment and survival in the tissues (refer to Figure 1) [15, 38–41]. Again, *in vitro* experiments demonstrated TSLP in presence of pro-inflammatory stimuli IL1-beta/tumor necrosis factor alpha (mimicking an ongoing inflammatory state) activated MCs to release IL-5 and IL-13; thereby, suggesting subsidiary mechanisms that produce IL-5 and can promote eosinophil numbers in the inflamed parenchyma/airway lumen [42]. IL-13 (and IL-4 in airway smooth muscle, ASM) trigger the release of eotaxins from the ASM [43] and the bronchial epithelium [44] that promote recruitment of eosinophils and eosinophil progenitors (refer to Figure 1). Increased IL-13 and IL-4 can promote the homing of CD34^+^ haemopoietic progenitor cells into the airway parenchyma [45].

Evidence suggest that this mechanism maybe upstream of activation by epithelial cell-derived cytokines [46, 47]. Understanding physiological processes that promote airway eosinophilia in severe asthma may be critical to the development of novel treatment modalities for optimal asthma control. Luminal eosinophilia in asthma arise as a result of (i) the recruitment of mature eosinophils from the periphery in response to locally elaborated chemo-attractants such as eotaxin and/or (ii) the localized maturation of eosinophil lineage-committed progenitors, termed “*in situ* differentiation” in the presence of locally elaborated cytokines such as IL-5 [48, 49]. That haemopoietic progenitors differentiate within the tissue is inferred from findings that there is increased recruitment of eosinophil progenitor cells into the airways in asthmatics [50]. In addition CD34^+^ cells extracted from human nasal polyp tissue and nasal explant tissue undergo IL-5 driven differentiation to form mature eosinophils [51, 52]. In context, anti-IL5 mAbs reduce tissue and luminal eosinophils (see Table 1), and are most effective in severe prednisone-dependent asthmatics with eosinophilic bronchitis [21]. These findings suggest that local eosinophilopoiesis may be a more dominant mechanism for the persistence of eosinophils in the airways of patients with moderate-to-severe asthma than chemokine-dependent (for e.g. eotaxin) recruitment of mature eosinophils. Whilst this remains to be determined, it may explain our recent findings where treatment with anti-CCR3 failed to clear luminal eosinophils likely because the treatment did not attenuate local differentiative processes [37].

Controlling the development of airway eosinophilia may involve targeting multiple factors that stimulate eosinophils recruitment and modulate local differentiative processes or prolonged tissue survival. A few conducted clinical trials with mAbs targeting IL-4 and IL-13 biology, similar to anti-IL-5, has met with a mixed response in improving clinical symptoms [10]. However, a combination therapy with drugs like Dupulimab (targets the receptor complex common both IL-4 and IL-13) [53] and an anti-IL-5 mAb could synergistically curb the mechanisms of *in situ* eosinophilia plausibly altered in severe asthmatics, that render the airways susceptible to maintain the clinical symptoms.

### Anti-IL-5 therapy for other lung eosinophilic disorders

As evident from the on-going discussion, IL-5 and IL-5 receptor alpha (IL5Rα) exhibit an undeniable eosinophil lineage-specificity. Indisputably, they have been considered as a potential therapeutic target in eosinophilic airway disorders.

### Hypereosinophilic syndrome (HES)

HES is a heterogeneous rare disorder defined by the presence of >1500 eosinophils per μL of blood, persistent for ≥ 6 months, with eosinophil-related organ involvement or dysfunction and no identifiable secondary cause of eosinophilia [5]. Early case studies with HES patients show considerable improvement in disease symptoms, patient relief, and decrease in eosinophilia with 750 mg intravenous Mepolizumab [5]. Table 2 summarizes an open-label study with 4 patients in 2004 [54] followed by a double-blinded, multi-centered clinical trial with Mepolizumab in 2008, where 41 out of 43 patients (on the experimental drug and tapering strength of prednisone), managed to maintain a circulating blood eosinophil count< 600/μL, for ≥8 weeks (p< 0.0001, *vs.* placebo) [55]. Additionally, it could be an alternative to using high-dose OCS, otherwise prescribed to the patients diagnosed with T-lymphocyte variant of HES [56]. A number of studies are currently underway to evaluate other anti-IL5 molecules in addition to Mepolizumab as potential therapeutic interventions in HES. A study with HES patients is projected to end in early 2017, which evaluates the safety and efficacy of Benralizumab (NCT02130882) in these subjects (http://clinicaltrials.gov).

**Table 2:** **Anti-IL5 trials in eosinophilic lung disorders**

First author [ref]/year/drug	Disease (severity)	Study design	Dosage/ delivery	Baseline eosinophil count	Comments on eosinophilia	Outcome summary
Garrett [54]/ 2003	HES	n= 4 open label	3 doses 10 mg/kg or 750 mg (max) i.v. every 4 wk	• Blood eos > 750/μL after an 8 wk pre-treatment run in period	• Blood eos reduced in all patients, sustained in 12 wk follow-up span	• Symptoms and quality of life improved in all patients
**Mepolizumab**	Severe, uncontrolled					
						• Progressive improvements in FEV_1_
Rothenberg [55]/2008	HES	n= 85 db, pc,, mc, parallel group study	750 mg i.v. at 4 wk interval 36 wk study	• Blood eos<1000/μL after a 6 week run-in period with prednisone therapy	• Blood eos<600/μl for 8 wks, achieved in 95% patients in drug group , 45% placebo, p< 0.0001	• Primary end-point (reduction of prednisone to 10 mg or less without clinical severity) was reached 84% of patients in drug group, 43% placebo, p< 0.0001
**Mepolizumab**	(patients negative for FIP1L1-PDGFRA fusion gene)					
				Baseline (median all patients):		
				• Blood eos (x 10^9^/L): 0.447 ± 0.694	• Sputum eos not measured	
Roufosse [56]/ 2010	L-HES	n=85 db, pc, international study	750 mg i.v. at 4 wk interval 36 wk study	• Controlled eosinophil levels (<1000/μL)by OCS monotherapy at a daily dose of 20–60 mg.	• Blood eos were maintained ≤ 600/μL by L-HES (Mepolizumab group) for 8 wks and during the entire length of the study compared to placeb0	• Significant lower mean daily prednisone dose of 4.64 mg in drug dosed group , compared to 28.3 mg in placebo (P=0.014)
**Mepolizumab**	(T-lymphocytic variant) – recruitment based on T-cell phenotyping and profile negative for FIP1L1-PDGFRA gene					
						• Patients with low CCL17 levels were seen to significantly maintain blood eos ≤ 600 μl
Kim [57]/2010	EGPA	n= 7	4 monthly 750 mg (i.v)	• Mean eos count 3.4%	• Reduction in eos count from 2.9% (mean) to 0.4 at wk 16 (wash-out phase)	• Mean reduction in corticosteroid 18.8 mg to 4.6 mg, P< 0.001
**Mepolizumab**	Mean FEV_1_ 76% predicted	open –label pilot study	40 wk study			
	Mean Prednisone dose 12.9 mg				• Eos mean 3.8% at wk 40	• Significant improvement of ACQ during study and wash-out phase
						• Patients clinically stable through study period, but EGPA manifestations on cessation of test drug
Moosig [58] 2011	Active refractory (n= 3) or relapsing (n= 7) active EGPA	sc, phase II, uncontrolled	750 mg i.v. once every 4 weeks (9 infusions in total)	• BVAS does not include eos as a criteria	• 6 patients (≥120 cells/μl) showed reduction in eos from their respective baseline, maintained throughout.	• Disease extent dropped from 4at weel 0 to 0 at week 32 (p= 0.009)
**Mepolizumab**						
	OCS ≥ 12.5 mg/daily			• Variations in eos levels ranged from 13 – 4282 cells/μl		• Eight patients achieved remission at week 32 (primary end-point), BVAS score= 0, OCS<7.5 mg/day
	BVAS ≥ 3					
						• No relapse occurred
Brightling [59] 2014	Moderate to severe	n= 101	100 mg s.c. every 4 weeks (three doses), then every 8 weeks (five doses) over 48 weeks	• Inclusion criteria Sputum eosinophils > 3% at screening or past year	• Significant reduction in both sputum and blood eosinophil levels at week 4, and maintained till week 56	• No changes in acute exacerbation rates, lung function or symptom score between treatment and placebo arm at week 56 for overall population
**Benralizumab**	COPD	Phase II				
	Exacerbations ≥ 1 in previous year	Mc, pb,db				
				• Sub-group analysis stratified results based on ≥ 150 or ≥ 200 or ≥ 300 eosinophils/μl	• Increase in blood and sputum eos after final dose	
						• non-significant decrease in exacerbation rate compared with placebo in patients with baseline eosinophil counts of ≥ 150 cells/ uL(p= 0°84), ≥ 200 cells/ uL (p= 0°26), or 300 cells/μl (p= 0°28)
						• Changes in FEV_1_ at week 56 was significant in patients with blood eosinophil counts ≥150 cells/ μL (p= 0°031) or ≥ 200 cells/ μL (p= 0°035), and non-significant in those with counts of ≥ 300 cells/μL (p= 0°22)

Index: eos= eosinophils; db= double-blind; pc= placebo-controlled; mc= multi-center; sc= single-centre; FEV1= peak expiratory flow i.v.= intravenous; s.c.= sub-cutaneous; wk= week; ns= non-significant; FIP1L1–PDGFRA :Fip1-like 1/platelet-derived growth factor receptor a fusion; ACQ= Asthma Control Questionnaire; Birmingham Vasculitis Activity score= BVAS.

### Eosinophilic Granulomatosis and Polyangitis (EGPA)

EGPA, earlier known as Churg-Strauss syndrome, is a rare form of vasculitis characterized by asthma and eosinophilia, with multi-organ involvement (lung, peripheral nerves, heart, gastrointestinal tract, skin), where systemic corticosteroid is the cornerstone of therapy [4, 60]. Histology shows classic evidence of an eosinophilic inflammatory response in the airway tissue of these patients and hence Mepolizumab has been reasoned to be a potential corticosteroid-sparing therapy. A recent case study reported complete regression of asthma (discontinuation of inhaled therapy) and depletion of blood and airway eosinophils in a patient with refractory EGPA with monthly infusions of 750 mg Mepolizumab [60]. Kim *et al*., 2010, reported a significant (75%) reduction in circulating eosinophils with 4 monthly doses of 750 mg (i.v) Mepolizumab, in a small open label trial with 7 patients, that allowed safe reduction of OCS from a mean dose of 18.8 mg to 4.6 mg [57]. In addition, Mepolizumab at the same dosage allowed complete remission in 8 out of 9 EGPA patients in a Phase II uncontrolled trial (detailed in Table 1) [58]. Though promising, further clinical investigations are necessary to ascertain the therapeutic benefit of Mepolizumab in EGPA and a large multicenter clinical trial is ongoing.

### Chronic eosinophilic pneumonia (CEP)

Chronic eosinophilic pneumonia (CEP) is an idiopathic condition that presents with peripheral eosinophilia, eosinophilic infiltrates in the lung parenchyma and may occasionally be associated with asthma. Increased levels of IL-5 and release of cytotoxic granular proteins from eosinophils constitutes an important pathomechanism in pulmonary tissue damage observed in CEP [61]. Conventionally OCS therapy is used for treatment; however, an eosinophil-targeted therapy with Mepolizumab might result in steroid-sparing therapeutic benefit in CEP patients.

### Chronic bronchitis (in COPD)

Chronic bronchitis is a primary component of COPD that encompasses a high level of heterogeneity. A sputum database analysis of 2443 patients with airway diseases, revealed one-fifth of the COPD patients experience eosinophilic bronchitis (EB). Additionally, EB was also associated with the severity of airflow obstruction in non-asthmatic COPD. 18% COPD patients with frequent exacerbations were documented to have EB and associated eosinophilia. Targeting eosinophils and IL-5 levels with Mepolizumab may decrease exacerbation rates and improve lung functions for this sub-set of COPD patients [62], as documented for other eosinophil-driven airway disorders (discussed previously). However, as per the recent reports of Brightling and co-workers, no reduction in annual exacerbation rates was observed in moderate to severe COPD patients with eosinophilia (>3% sputum eosinophils) when treated with a monthly/bimonthly subcutaneous dose of 100 mg Benralizumab, even though the treatment effectively depleted both airway and circulating eosinophils (refer to Table 2) [59]. Nevertheless, online database (http://clinicaltrials.gov, last accessed 16/09/2014) enlists ongoing independent studies investigating efficacy and safety of Mepolizumab as an adjunct treatment in COPD management (registration number: NCT02105961); in COPD with EB (NCT01463644); reducing exacerbations in severe COPD (NCT02105948); and Phase III trials for Benralizumab in moderate to very severe COPD (NCT02155660, NCT02138916), addressing safety and efficacy of the drug.

## Summary

The ongoing anti-IL5 clinical trials, show promise of a safe, effective treatment option for the severe ‘eosinophilic’ asthma endotype independent of their atopic status [63]. Beyond asthma, small pilot studies have documented their potential in treating HES and EGPA patients. IL-5 may not be the sole determinants of persistent airway eosinophilia. Recently described immune cells such as the ILC_2_s and epithelium-derived type-2 alarmins that release T_H_2 cytokines like IL-13 may also play important rolesHence, targeting IL-5 and IL-13 to curb the eosinophil-derived clinical symptoms needs to be investigated in select patient-subtypes. Finally, while blood eosinophil count or other indirect assessments such as the eosinophil/lymphocyte ratios may help to identify an “eosinophilic phenotype” to initiate therapy with an anti-eosinophil biologic drug in patients with moderate asthma, this strategy may not be as effective as measuring sputum eosinophils to monitor response to therapy particularly in more severe systemic corticosteroid-dependent asthmatic patients. The key to successful therapy would be to select the appropriate patient population. The mere presence of eosinophils in blood or sputum may not be sufficient. The patients who are likely to respond are those whose disease is truly largely dependent on eosinophil biology. Identification of these patients require clinical acumen, clinical criteria and demonstration of persistent (not transient) systemic and airway eosinophilia.

## Abbreviations

mAb: Monoclonal antibodies
IL5Rα: Interleukin-5 receptor alpha
HES: Hypereosinophilic syndrome
EGPA: Eosinophilic granulamatosis and polyangitis
COPD: Chronic obstructive pulmonary disorder
FEV_1_: Forced expiratory volume in 1 second
ACQ: Asthma control questionnaire
OCS: Oral corticosteroid
IL: Interleukin
ECP: Eosinophilic cationic protein
CCR: Chemokine chemokine receptor
TSLP: Thymus stromal lymphopoeitin.
